# Structural modulation of gut microbiota during alleviation of non-alcoholic fatty liver disease with *Gynostemma pentaphyllum* in rats

**DOI:** 10.1186/s12906-020-2835-7

**Published:** 2020-02-05

**Authors:** Shu-Hua Shen, Ting-Yan Zhong, Cui Peng, Jie Fang, Bin Lv

**Affiliations:** 1 Department of Healthcare Management, The First Affiliated Hospital of Zhejiang Chinese Medical University, Hangzhou, 310006 China; 2 School of Life Sciences and Biotechnology, Shanghai Jiao Tong University, Shanghai, 200240 China; 3 Laboratory Animal Centre, Zhejiang Academy of Medical Science, Hangzhou, 310000 China; 4 Department of Gastroenterology, The First Affiliated Hospital of Zhejiang Chinese Medical University, 54 Youdian Road, Zhejiang, 310006 Hangzhou China

**Keywords:** Non-alcoholic fatty liver disease, *Gynostemma pentaphyllum*, Gut microbiota, Endotoxemia, Insulin resistance

## Abstract

**Background:**

The current work aimed to assess whether *Gynostemma pentaphyllum (GP)*, a Chinese herbal medicine, structurally modifies the gut microbiota in rats during non-alcoholic fatty liver disease (NAFLD) treatment.

**Methods:**

High-fat diet (HFD)-induced NAFLD rats were orally administered water decoction of *GP* or equal amounts of distilled water per day for 4 weeks. Liver tissues were examined by histopathological observation, while intestinal tissues were examined by both histopathological and ultrastructural observations. The levels of fasting blood glucose (FBG), fasting serum insulin (FINS), total cholesterol (TC), triglycerides (TG), high-density lipoprotein cholesterol (HDL-C), low-density lipoprotein cholesterol (LDL-C), alanine transaminase (ALT) and aspartate transaminase (AST) were measured by enzymatic method. The levels of toll-like receptor 4 (TLR-4), tumor necrosis factor-alpha (TNF-α), interleukin-1-beta (IL-1β) and interleukin-6 (IL-6) in both serum and hepatic tissues were measured by RT-qPCR. The protein expression level of TLR-4 in hepatic tissues was detected by western blot. The gut microbiota was assessed by 16S rRNA-based microbiota analysis.

**Results:**

*GP* maintained intestinal integrity and reversed gut dysbiosis in high-fat diet (HFD)-induced NAFLD rats. This also reduced the ratio of *Firmicutes* to *Bacteroidetes*, enriching the abundance of beneficial bacteria (*Lactococcus spp*.) and inhibiting the abundance of pathogenic bacteria (*Ruminococcus spp*.) in the gut. The levels of pro-inflammatory cytokines (TNF-α, IL-1β and IL-6) and the expression of TLR4 were downregulated (*P < 0.05*), while the insulin resistance index, HOMA-IR showed improvement by *GP* treatment (*P < 0.05*). Liver function indicators (ALT and AST) were remarkably decreased (*P < 0.01*). Besides, *GP* treatment reduced TG and LDL-C levels (*P < 0.05*), and increased HDL-C level (*P < 0.05*) compared with NAFLD group.

**Conclusion:**

The structural alterations of gut microbiota induced by *GP* are associated with NAFLD alleviation.

## Background

Nonalcoholic fatty liver disease (NAFLD) is defined as the presence of significant hepatic lipid accumulation (at least in 5% of hepatocytes) in the absence of competing liver disease etiologies, including chronic viral or autoimmune hepatitis, Wilson’s disease, and heavy alcohol consumption [1, 2]. NAFLD represents a group of ailments, ranging between simple steatosis and non-alcoholic steatohepatitis (NASH), which at times lead to cirrhosis [3]. It is considered one of the main factors causing liver disease worldwide, with a prevalence of around 25–45% [4]. In addition, NAFLD is increasingly recognized as the liver disease component of metabolic syndrome (MetS) [5], and is associated with a broad spectrum of diseases including obesity, type 2 diabetes (T2D), hyperlipidemia, hypertension, cardiovascular disease (CVD), and cancer [6, 7]. As a result, NAFLD patients have an increased risk for both liver and MetS morbidity and mortality [8], causing substantial economic and clinical burden to society [9].

NAFLD pathogenesis remains incompletely understood. The “multiple hit” hypothesis provides a relatively accurate explanation, wherein multiple insults act jointly in individuals with genetic predisposition to trigger NAFLD as well as associated complications. Such hits include insulin resistance, nutrition, gut microbiome, hormones secreted by the fat tissues, and genetic and epigenetic factors [10]. Recently, considerable evidence suggests gut microbiome dysbiosis has a critical function in NAFLD progression [11]. Dysbiosis increases gut permeability to bacterial products, promotes energy absorption, aggravates insulin resistance (IR), facilitating systemic bacterial translocation and hepatic inflammation [12].

Traditional Chinese Medicine has been employed in Asia for many thousand years [13]. Much attention has been paid to its remarkable efficacy and low side effects in treating MetS, especially NAFLD [14, 15]. Gynostemma pentaphyllum (*GP*), one of the popular herbs, has been used in traditional medicine since ancient times in treating various diseases, such as hyperlipemia, hyperglycemia, hepatitis, gastroenteritis [16]. We and others have demonstrated that *GP* has functions in protecting against NAFLD and other components of MetS [17, 18]. Cell culture and animal models have indicated the important role of *GP* in lipid reduction, glucose regulation [19, 20], as well as liver protection [21, 22]. Our preliminary study in high-fat diet (HFD) rats demonstrated that *GP* can effectively reduce blood lipid levels and protect liver function, negatively correlating with the content of gypenoside (saponins isolated from *GP*). However, it remains unclear regarding the mechanism of how *GP* alleviates NAFLD.

Hence, in this study, we explored the effect of *GP* on NAFLD (HFD induced) rats using dilinoleoyl phosphatidylcholine (*DLPC*) as a contrast [23, 24].

## Methods

### Drug preparation

**Fig. 1:**
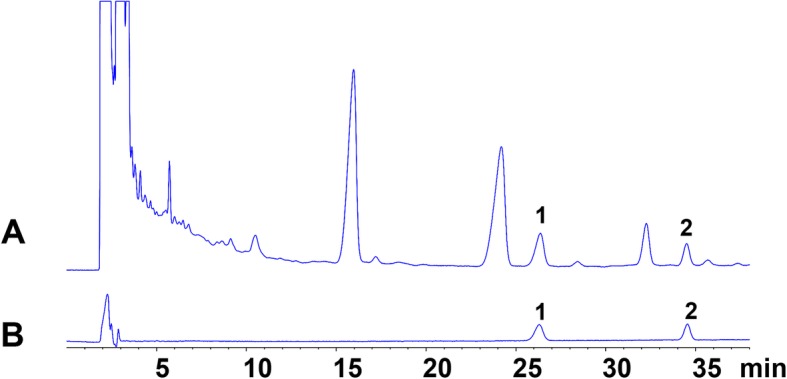
High-performance liquid chromatography chromatogram of sample (**a**) and standard (**b**) solutions. 1: Ginsenoside Rb3, 2: Ginsenoside Rd.

The *GP* material was obtained from the First Affiliated Hospital of Zhejiang Chinese Medical University (purchased from Huadong Medicine Co., Ltd., Date of Production: 20160706, Hangzhou, China) and identified by the Director of traditional Chinese pharmacy of the hospital, Mrs. Wen-Xia Zheng. *GP* was decocted twice with 4000 ml deionized water, 1 h each time, followed by concentrating it to 2 g per milliliter (2 g/ml) under normal pressure and making into a liquid extract. The quality of the *GP* was controlled by HPLC -MS analysis (Fig. 1). The collection, processing and usage of *GP* were performed according to the Guidelines for clinical use of the Pharmacopoeia of the People’s Republic of China.

### Animal experiments

#### Experimental animals

The rats were bought from Zhejiang Laboratory Animal Center (Production License Number: SCXK (Zhe)2014–0001, Use License Number: SYXK (Zhe)2014–0008). Animal experiments were approved and performed in accordance with the guidelines of the Ethics Committee of the first affiliated hospital of Zhejiang Chinese medical University, Hangzhou, China (Approval Number: 2017-k-098). Sixty male adult pathogen-free Sprague-Dawley (SD) rats, weighing 180-220 g were selected for this study. The rats were randomly divided into control group (*n* = 10, Control), model group (*n* = 10, NAFLD), positive drug group (*n* = 10, DLPC), high dosage group (*n* = 10, GPH), middle dosage group (*n* = 10, GPM) and low dose group (*n* = 10, GPL).

#### Molding

The rats were acclimatized to the housing conditions for 7 days, with free access to water and standard chow diet in laboratory conditions (18–24 °C, day-night cycle of 12 h with light changes at 6:00 am and 6:00 pm). After that, the rats were fed with chow diet or high-fat diet for another 4 weeks before drug administration. Animals in the control group were administered with standard chow consisting of 67% carbohydrate, 10% fat, 23% protein, providing a total calorie of 3.6 kcal/g. While the animals in the model group, the positive drug group and the high, middle and low dosage groups were continuously fed on HFD consisting of 52% carbohydrate, 30% fat, 18% protein, which provided a total calorie of 4.8 kcal/g [25]. According to our previous studies and preliminary experiments, indices such as body weight, intake and activities, fasting blood glucose (FBG), total cholesterol (TC), triglycerides (TG), high-density lipoprotein cholesterol (HDL-C), low-density lipoprotein cholesterol (LDL-C), alanine transaminase (ALT) and aspartate transaminase (AST) to confirm the success of model establishment*.*

#### Drug administration

From week 5, the *GP* treatment groups were orally administered water decoction of *GP* at concentrations of 6 g·kg^− 1^·d^− 1^, 3 g·kg^− 1^·d^− 1^ and 1.5 g·kg^− 1^·d^− 1^ once a day for another 4 weeks based on the conversion formula between human per 70 kg and rats per 200 g (1: 0.018) and our preliminary experiments. The rats in the positive drug group were orally administered *DLPC* at a concentration of 22.8 mg·kg^− 1^·d^− 1^, while the control group and the model group were administered equal amounts of distilled water per day for 4 weeks.

#### Specimen collection

After 8 weeks of HFD, the animals were fasted for 8 h. Next day, blood was drawn from the caudal vein, and then the rats were euthanized by cervical dislocation. The blood was centrifuged at 3000 rpm for 15 min, and the serum was stored at − 80 °C. After laparotomy, the liver, the contents from the cecum and the ileum and the colon besides the cecum were removed. The contents (about 1.0 g) of the cecum from each rat were taken, maintained in tubes with glass beads, and stored at − 80 °C. Part of the liver and the colon and terminal ileal tissues were fixed with neutral formaldehyde, and then the paraffin slices were prepared. Others were stored at − 80 °C.

#### Biochemical analyses

Biochemical assessments of serum lipids, glucose, enzymes, insulin and cytokines were performed in the central laboratory (The first affiliated hospital of Zhejiang Chinese medical University, Hangzhou, China).

The index of lipids in the serum, including TC, TG, HDL-C and LDL-C and the marker enzymes for liver damage and disease, including ALT and AST were measured by commercial enzymatic kits (Jiancheng Bioengineering Inst., Nanjing, China).

The index of fasting serum insulin (FINS), tumor necrosis factor-alpha (TNF-α), interleukin-1-beta (IL-1β) and interleukin-6 (IL-6) levels were measured by commercial ELISA kits (MultiSciences, Shanghai, China).

FBG was determined with a glucose meter (Sinocare, Changsha, China) by collecting the blood from the tip of the tail vein. Homeostasis model assessment of insulin resistance (HOMA-IR) was calculated using the formula: fasting glucose× fasting insulin/22.5 [26].

### Histopathology observation

#### HE staining

Liver or intestinal mucosal specimens were formalin-fixed, paraffin-embedded, and cut into sections (4-μm thickness), which underwent staining with hematoxylin (Servicebio, Wuhan, China; 5 min) and eosin (Servicebio; 2 min). A light microscope (Nikon, Tokyo, Japan) was employed for observations, with 8 sections assessed per rat.

#### ORO staining

Liver cryosections (thickness, 6 μm) underwent staining with ORO (Servicebio) for 20 min, and then counterstained with hematoxylin (Servicebio) for 1 min. A light microscope (Nikon) was employed for observations, with 8 sections assessed per rat.

#### Ultrastructural observation

Intestinal mucosal sections were fixed in 2.5% glutaraldehyde (Sinopharm Chemical Reagent Co., Ltd., Shanghai, China) for 24 h and 1% osmic acid (Sinopharm Chemical Reagent Co., Ltd) for 2 h, dehydrated through a graded series of ethanol (Sinopharm Chemical Reagent Co., Ltd) and acetone solution (Sinopharm Chemical Reagent Co., Ltd), embedded in Epon812 (Servicebio), sectioned with ultramicrotome (Leica, Weztlar, Germany), and stained with uranyl acetate (Sinopharm Chemical Reagent Co., Ltd) followed by lead citrate (Sinopharm Chemical Reagent Co., Ltd), then observed with transmission electron microscopy (Hitachi, Tokyo, Japan). A total of 20 tissue sections were analyzed for each animal.

#### Real-time quantitative reverse-transcription PCR

Fifty mg liver tissue was weighed, and then the mRNA expression levels of toll-like receptor 4 (TLR-4), TNF-α, IL-1β and IL-6 were detected by quantitative real-time reverse-transcription PCR (Additional file 1: Table S5) according to the previously published RT-qPCR methods [27].

#### Western blotting (WB)

Twenty mg liver tissue was weighed, and the protein expression level of TLR-4 was detected by WB as described previously [28].

#### Gut microbiota analysis

Fecal specimens upon snap-freezing in liquid nitrogen were stored at − 80 °C. DNA extraction was carried out by the CTAB protocol. For cecal fecal specimens, the 16S rRNA gene encompassing V3–V5 regions, 18S rRNA V4 and V9 regions, ITS1 and ITS2 regions underwent amplification with Phusion® High-Fidelity PCR Master Mix in GC Buffer (New England Biolabs). TruSeq® DNA PCR-free sample preparation kit was used to prepare a library. Microbial sequencing was performed on the HiSeq2500 Illumina platform (PE250). High quality reads were obtained (QIIME V1.9.1) and underwent clustering into OTUs on the basis of 97% sequence similarity (Uparse v7.0.1001). The nearest alignment space termination multi-aligner was employed for aligning high-quality sequences based on SILVA compatible database alignment, removing non-aligned reads. Chimeric sequences detected by the UCHIME algorithm were also excluded. Read classification used a Bayesian classifier based on the RDP database generated by our team. We removed all reads not classifiable at the kingdom level. For alpha diversity assessment, rarefaction analysis and Shannon index calculation were carried out with QIIME. Fast UniFracPCoA was carried out via phylogenetic tree construction and by inserting the representatives of various OTUs, also with QIIME [29]. As proposed previously [30], whether separation among animal groups in the principal coordinate analysis (PCoA) score plot is statistically significant was evaluated by multivariable analysis of variance test with statistically significant differences in physiological/biochemical parameters.

### Statistical analysis

Ten-replicate assays were presented as mean ± standard deviation (SD). Differences in biochemical indicators were examined by unpaired two-tailed Student’s t-test. Multiple groups were compared by one-way ANOVA with Newman–Keuls post hoc test. Next-generation sequencing data were examined by the Tukey’s test. *P* < 0.05 indicated statistical significance. In figures, different superscript letters indicate significant differences (post hoc ANOVA). R 3.4.3 was used for statistical analysis.

## Results

### GP prevents HFD-induced hyperlipidemia of NAFLD

Previous studies showed that the rats fed a HFD produced high levels of TC, TG and LDL-C, while the production of HDL-C was reduced [31, 32]. Using a rat NAFLD model, we observed that the rats fed on HFD for 8 weeks led to significant differences in the lipids levels when compared with control group (Table 1). TC, TG and LDL-C levels were significantly increased in the serum of NAFLD rats when compared with control rats, whereas the HDL-C level was significantly reduced. Meanwhile, for the middle dose and high dose *GP* treated rats, the serum levels of TC, TG and LDL-C were also reduced significantly when compared with NAFLD rats. Significant increase in HDL levels was observed in the medium dose of *GP* (GPM) and high dose of *GP* (GPH) rats. *GP* treatment demonstrated a dose-dependent effect, alleviating hyperlipidemia. *DLPC* treatment reduced the serum TG and LDL-C levels, and increased HDL-C level as expected. These findings suggested that *GP* prevents HFD-induced hyperlipidemia in rats (Table 1).

**Table 1:** *GP* prevents HFD-induced hyperlipidemia

	TC (mmol/L)	TG (mmol/L)	LDL-C (mmol/L)	HDL-C (mmol/L)
NAFLD	2.14 ± 0.26	0.63 ± 0.04	1.61 ± 0.10	0.32 ± 0.17
Control	1.26 ± 0.24**	0.50 ± 0.07**	1.26 ± 0.34*	0.47 ± 0.15**
DLPC	1.98 ± 0.62	0.51 ± 0.08*	1.15 ± 0.25**	0.47 ± 0.21*
GPL	1.87 ± 0.33	0.48 ± 0.07**	1.14 ± 0.21**	0.39 ± 0.12
GPM	1.69 ± 0.28*	0.48 ± 0.07**	1.16 ± 0.36**	0.51 ± 0.08**
GPH	1.29 ± 0.27**	0.49 ± 0.16**	1.14 ± 0.24**	0.54 ± 0.09**

Serum concentrations of TC, TG, LDL-C and HDL-C in each group compared with NAFLD group (*n* = 10 for each group). Data are shown as mean ± standard deviation. Whereas * and ** represent statistically significant results (*P* < 0.05, *P* < 0.01, respectively) based on Newman–Keuls post hoc one-way ANOVA analysis. GPL: low dose of *GP* treatment; GPM: middle dose of *GP* treatment; GPH: high dose of *GP* treatment

### GP prevents HFD-induced non-alcoholic fatty liver (NAFLD)

**Fig. 2:**
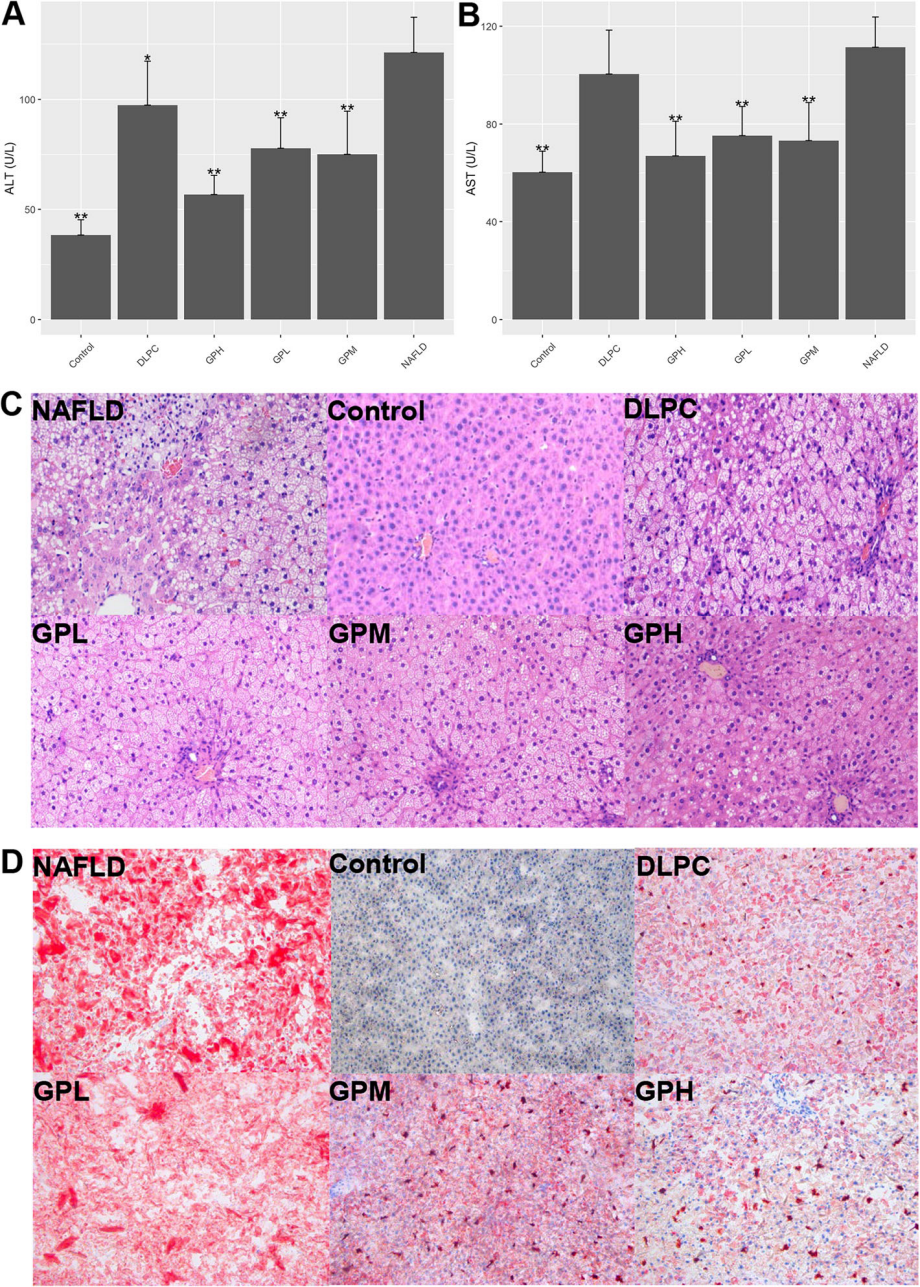
*GP* prevents HFD-associated Non-alcoholic fatty liver disease (NAFLD) in the rat model. **a**, (**b**) Serum concentrations of ALT and AST in various groups compared with the NAFLD group (*n* = 10 for each group). Data are mean ± standard deviation. *P < 0.05; **P < 0.01 (one-way ANOVA with Newman–Keuls post hoc analysis). **c** H&E staining of the liver tissue in the NAFLD, Control, DLPC, GPL, GPM and GPH groups (magnification, 200×) (*n* = 10 for each group). Major histopathological changes induced by HFD in rat liver included hepatosteatosis, ballooning and hiver inflammation. **d** Liver lipid content was determined by Oil-red O staining in the NAFLD, Control, DLPC, GPL, GPM and GPH groups (magnification, 200×) (*n* = 10 for each group)

Previous studies have shown that NAFLD rats produced high levels of marker enzymes for liver damage and disease, which included ALT and AST [31, 32]. The serum levels of the marker enzymes were measured. The results showed that ALT and AST in NAFLD rats were higher when compared with control rats. While *GP* treatment reduced both ALT and AST levels significantly. *DLPC* improved ALT to some extent, but showed no remarkable effect on AST (Fig. 2a, b, Additional file 1: Table S1).

As expected, both H&E staining and ORO staining revealed the normal structure of liver in control rats, while hepatic steatosis in NAFLD rats [31, 32]. However, the extent of steatosis in the liver of *GP* treated rats was remarkably reduced in a dose-dependent manner. *DLPC* also alleviated hepatic steatosis to a certain extent (Fig. 2c, d). These findings suggested that *GP* prevents HFD-induced NAFLD in rats.

### GP reduces inflammation, endotoxemia and insulin resistance

**Fig. 3:**
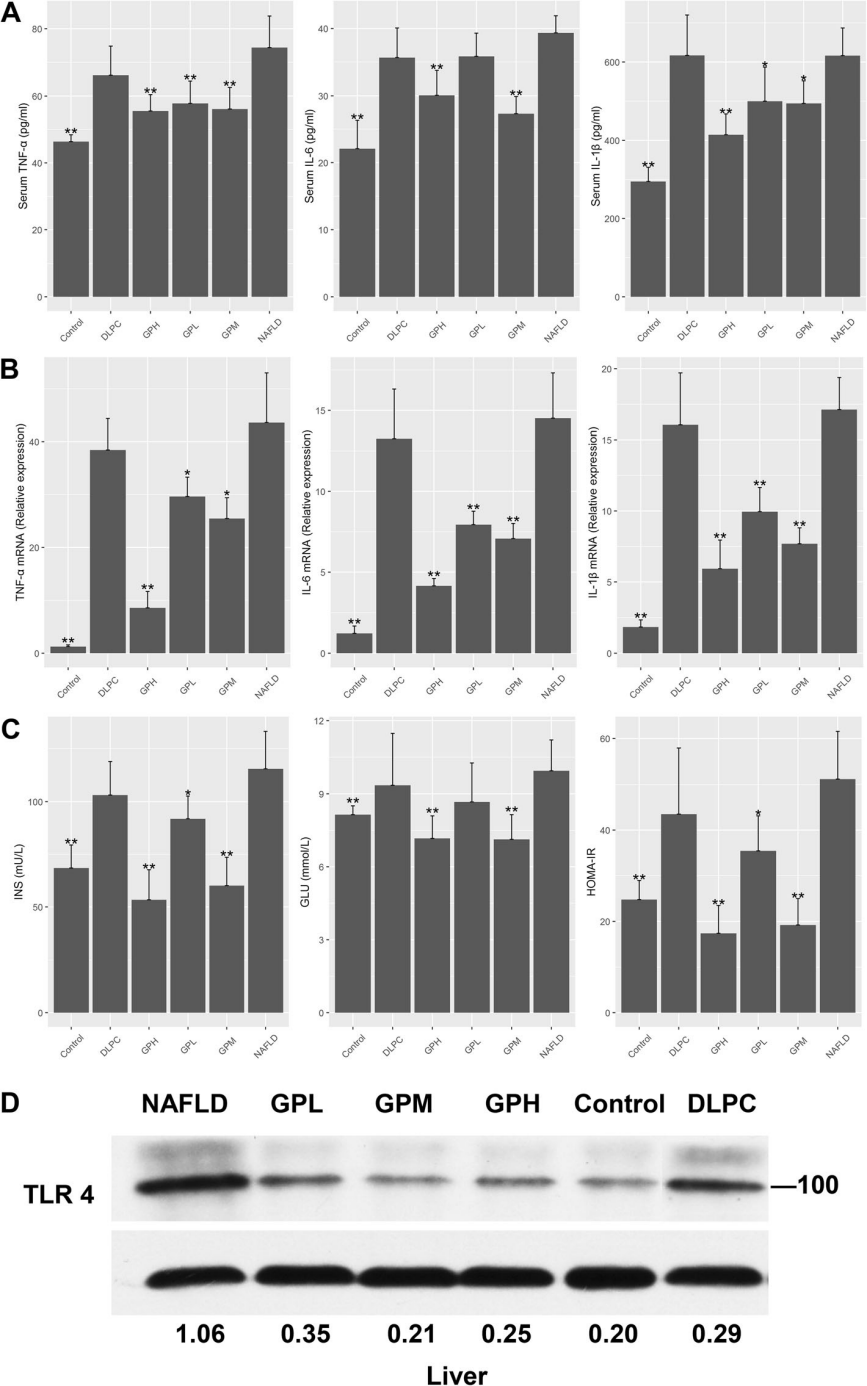
*GP* prevents HFD-induced inflammation, endotoxemia and insulin resistance in rats. **a** Serum TNF-α, IL-1β and IL-6 amounts in each group were compared with those of the NAFLD group (*n* = 10 for each group). **b** TNF-α, IL-1β and IL-6 mRNA levels in hepatic tissues as assessed by RT-qPCR, compared with the NAFLD group (*n* = 10 for each group). **c** Serum concentrations of INS, GLU and HOMA-IR in each group were compared with those of the NAFLD group (*n* = 10 for each group). Data are mean ± standard deviation. *P < 0.05; **P < 0.01 (one-way ANOVA with Newman–Keuls post hoc analysis). **d** Protein amounts were normalized to Actin expression, and the relative ratios based on the Control group are shown below the immunoblots

Previous studies have shown that NALFD rats produce high amounts of pro-inflammatory cytokines in the liver and serum, e.g., TNF-α, IL-1β and IL-6 [33]. We measured the above cytokines in rats following 8 weeks of HFD with or without *GP* or *DLPC* treatment. The results showed that TNF-α, IL-1β and IL-6 amounts were higher in serum and hepatic tissues of NAFLD rats compared with control animals. Moreover, *GP* treatment resulted in decreased TNF-α, IL-1β and IL-6 amounts, while *DLPC* administration did not (Fig. 3a, b, Additional file 1: Table S2,S3).

“Metabolic endotoxemia” may promote inflammatory reactions and insulin resistance in HFD-fed rats through TLR4 signaling [34, 35]. We examined the effects of *GP* and *DLPC* on TLR4 protein amounts in the liver. Insulin resistance was estimated using HOMA-IR. The results showed that *GP* reduced endotoxemia and insulin resistance in HFD-fed rats, while *DLPC* did not (Fig. 3c, d, Additional file 1: Table S4). These findings suggested that *GP* reduced inflammation, endotoxemia and insulin resistance in NAFLD rats.

### GP maintains intestinal integrity

**Fig. 4:**
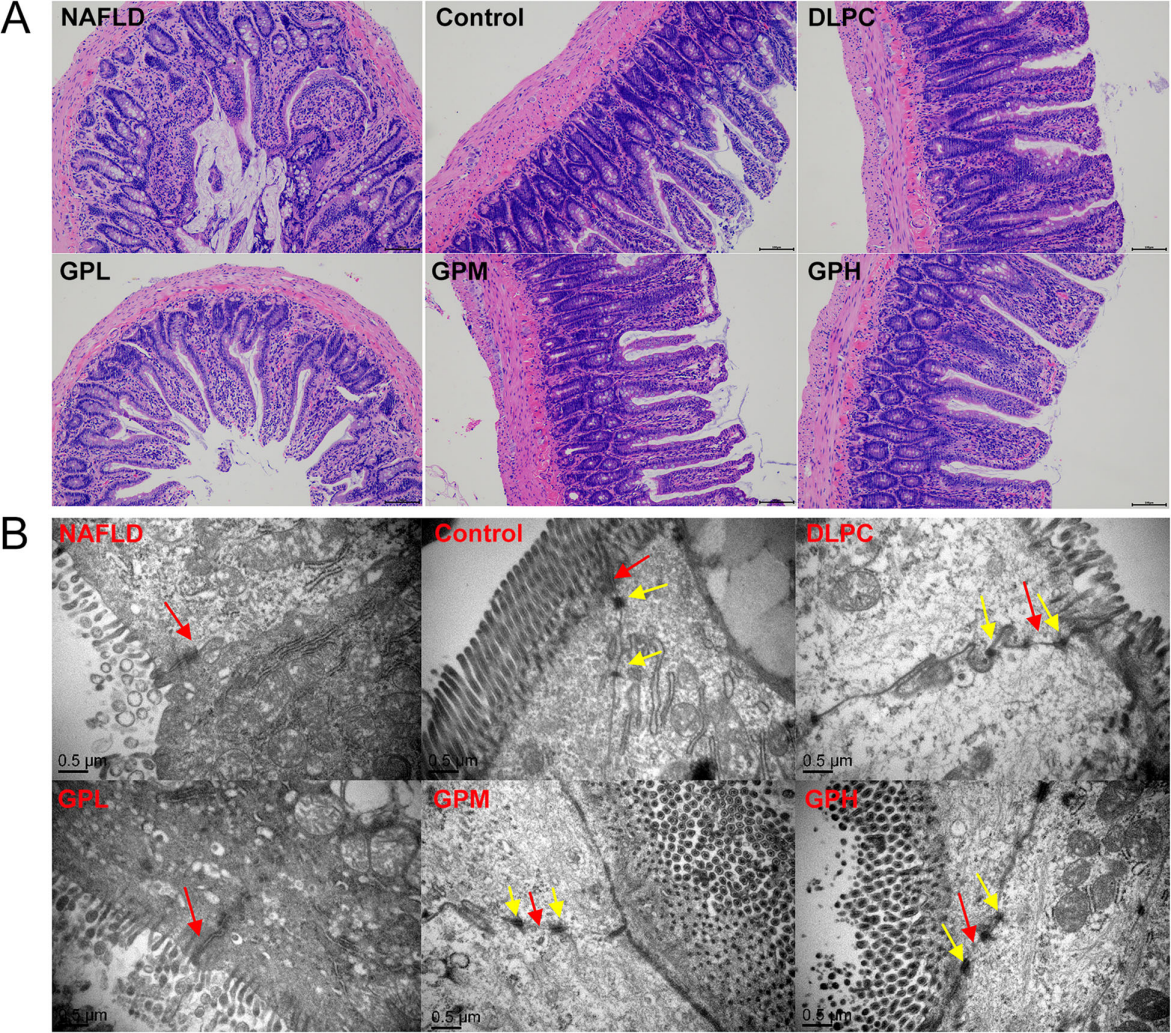
*GP* maintains intestinal integrity in rats. **a** HE staining of ileal tissue in NAFLD, Control, DLPC, GPL, GPM and GPH groups (magnification, 100×) (*n* = 10 for each group). Major histopathological changes induced by HFD in rat ileum were sparse and irregular microvilli of epithelial cells with inflammatory cell infiltration, damaged tight junctions and desmosomes. **b** Transmission electron microscopy of ileum in NAFLD, Control, DLPC, GPL, GPM and GPH groups (magnification, 30,000×) (*n* = 10 for each group). Red arrow: tight junctions; Yellow arrow: desmosomes

Given that gut microbiota dysbiosis in HFD-fed rats might alter intestinal permeability and promote lipopolysaccharide (LPS) release into the blood stream [36], whether *GP* and *DLPC* modulate intestinal barrier integrity was investigated. In the control group, the microvilli of epithelial cells were rich and regular, and the tight junctions were clear and complete, with small gaps and abundant desmosomes. In the NAFLD group, the microvilli of epithelial cells were sparse and irregular, and the tight junctions were damaged, with large gaps and loose or few desmosomes. Compared with NAFLD group, the rats in *GP* and *DLPC* groups showed more regular microvilli, improved tight junctions and desmosomes, and smaller gaps. The former ones were dose-dependent, showing a better result than the latter (Fig. 4a, b). These findings suggested that *GP* might improve intestinal barrier integrity in NAFLD rats.

### The gut microbiome is structurally modulated by GP administration

**Fig. 5:**
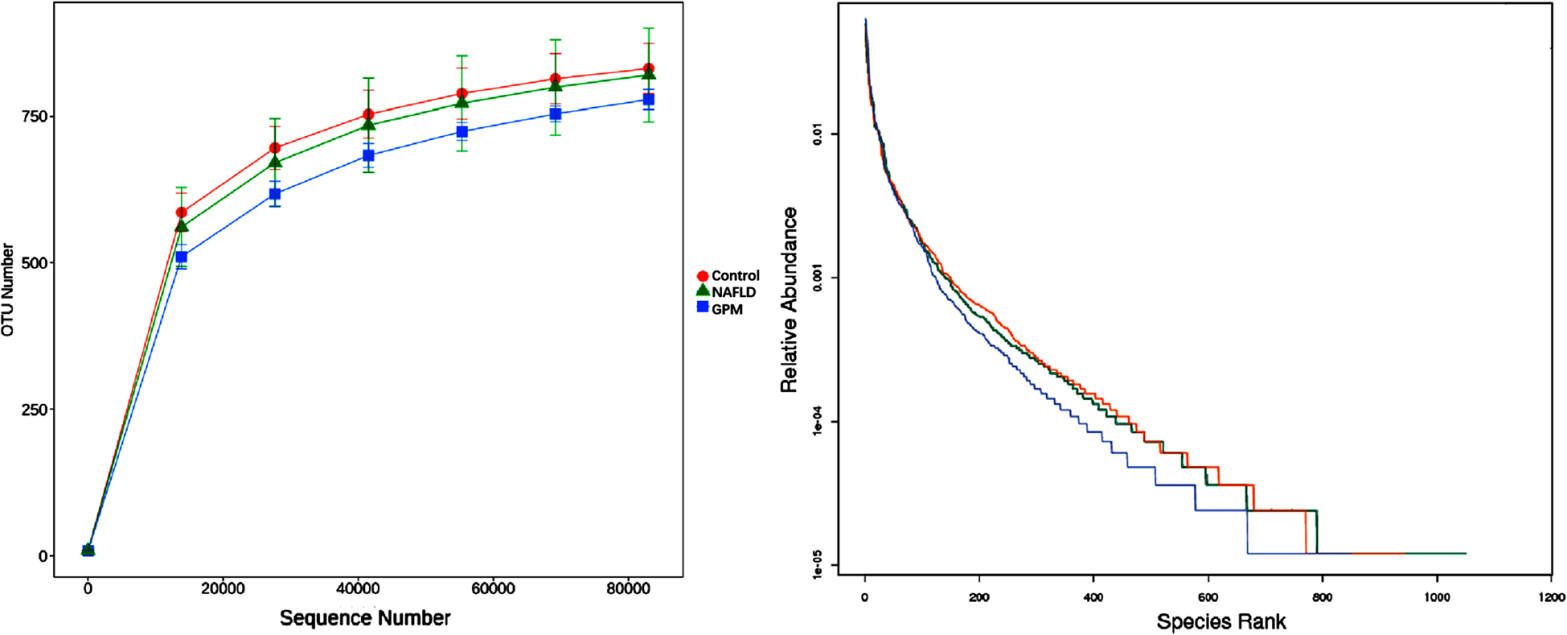
Rarefaction diversity and Rank abundance curves. We examined the structural alterations of the gut microbiome in the NAFLD, Healthy control and *GP* middle dose groups, respectively, before and after *GP* treatment. Rarefaction diversity and Rank abundance curves showed that although rarefaction diversity curves showed no plateau, the diversity was mostly captured, indicating that the sequencing results could represent the bacterial diversity of the gut

**Fig. 6:**
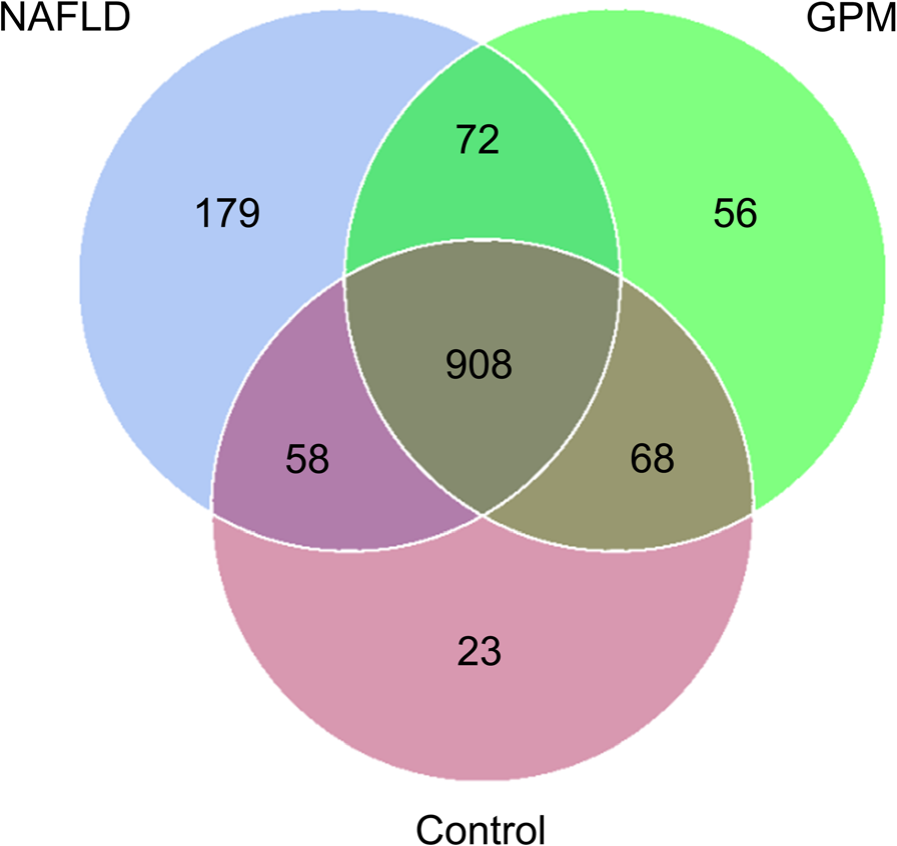
Venn plot of the number of OTUs in each group

**Fig. 7:**
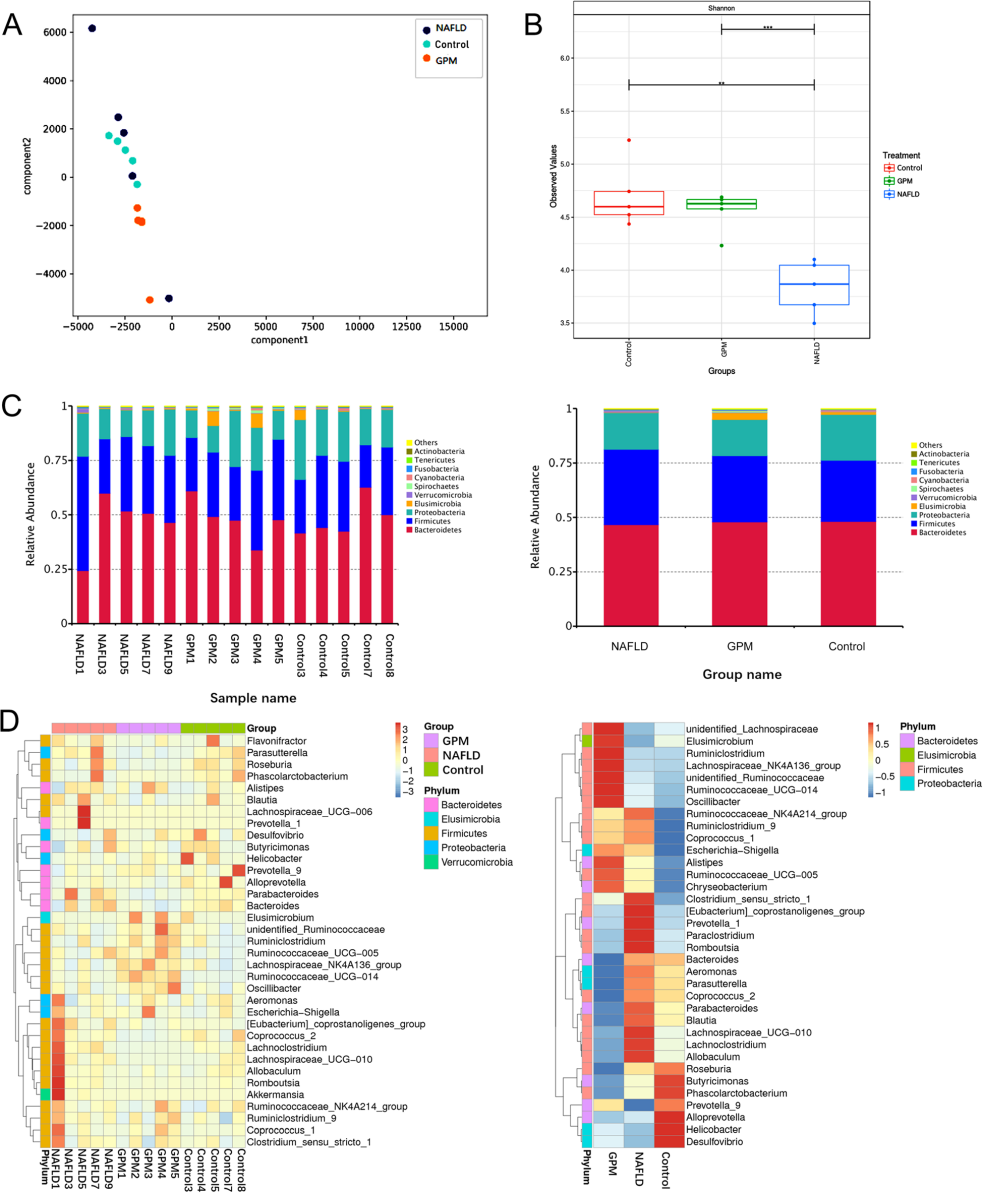
*GP* alters microbiome composition in HFD-fed rats. Microbiome composition in fecal specimens from chow-fed and HFD rats administered *GP* or not were assessed by next generation sequencing (*n* = 5/group). **a** PCA plots for various groups. **b** Shannon indexes. **c** Taxonomic profiles at the phylum level of gut bacteria in various groups. **d** Heat map reflecting the abundance levels of 35 OTUs representing the bacterial taxa significantly affected by *GP* in HFD-fed rats. Red and blue colors indicate OTU level increase and decrease, respectively, in different groups

We next examined gut microbiome compositions in three animal groups, including the NAFLD, healthy control and *GP* middle dose groups, pre- and post-*GP* administration, respectively. A total of 750,000 acceptable raw sequences (34,753 unique sequences) and 3222 OTUs were detected in 15 samples, with averagely 874 ± 101/sample. Although rarefaction diversity curves showed no plateau, the diversity was mostly captured (Fig. 5). PCA analysis revealed that compared with the HFD-induced NAFLD model group, the *GP* treatment group showed closer deviation towards the healthy animals, suggesting that gut microbiota structure in rats showed divergence from baseline after a 4-week *GP* treatment (Fig. 7a). The Venn diagram indicated that *GP* treated and control rats shared more OTUs than NAFLD rats (Fig. 6). Unique OTUs in NALFD rats were triple the amounts in *GP* treated rats (Fig. 6). Shannon index analysis showed that intestinal flora density in the NAFLD group was significantly reduced, and *GP* treatment resulted in recovered intestinal flora diversity compared with control rats (Fig. 7b). The gut microbiota of rats consisted of *Bacteroidetes*, *Firmicutes*, *Proteobacteria*, *Elusimicrobia*, *Cyanobacteria*, *Fusobacteria*, *Actinobacteria*, *Spirochaetes* and *Verrucomicrobia* at the phylum level. Of these, *Bacteroidetes* were the most abundant organisms, followed by *Firmicutes* and *Proteobacteria* (Fig. 7c). Several studies have indicated that elevated *Firmicutes* to *Bacteroidetes* (two main phyla) ratio is associated with lipid metabolism disorder [37–39]. Lipid metabolism has an important function in NAFLD [40]. Namely, *GP* treatment (GPM) decreased the *Firmicutes*-to-*Bacteroidetes* ratio in HFD-fed (NAFLD) rats to a value comparable to that of control rats (Fig. 7d). The abundance levels of *Elusimicrobia* and *Cyanobacteria* were both higher in *GP* and control rats in comparison with HFD-fed rats (Fig. 7d). Besides, many bacterial species showed increased amounts after *GP* administration in comparison with the model group, indicating that *GP* may enrich specific bacterial species (Fig. 7d). Moreover, *GP* treatment increased the abundance levels of beneficial bacteria such as *Lactococcus sp*p. and decreased those of the pathogenic bacteria, including *Ruminococcus spp*. (Fig. 7d). Collectively, these results showed that *GP* modulated the gut microbiome in HFD-fed rats, yielding a microbiome composition comparable to that of control rats.

## Discussion

Traditional Chinese Medicine has been used for treating several diseases for thousands of years in China. Nevertheless, Chinese herbs are highly complex with undefined mechanisms, which prevents the identification of active chemical components. Many studies have suggested that Chinese herbs prevent or alleviate diseases by controlling gut microbiome structure [41–44]. This research may provide a potential mechanism as to how *GP* alleviates NAFLD.

As shown above, *GP* administration exerted concentration-dependent effects in HFD-induced NAFLD rats. *GP* treated rats showed significant improvement in liver health by lowering ALT and AST levels, and decreasing hepatic steatosis. Besides, we observed an improvement in hyperlipidemia and hyperglycemia in *GP* treated animals, corroborating a previous Australian study [45]. These data showed that *GP* can alleviate NAFLD and other components of MetS, making it a promising candidate medicine for NAFLD and the associated complications.

The current model of HFD-induced NAFLD is largely explained by gut microbiota dysbiosis [46]. Pathogenic bacteria are excessively grown, producing too much LPS that exceeds hepatic clearance capacity, and therefore, “metabolic endotoxemia” [47]. After activation by LPS, TLR4 stimulates the production of inflammatory kinases (such as JNK, IKK and p38), inhibits the phosphorylation of insulin receptor substrates, and damages the insulin signal transduction pathway, resulting in the development of IR and inflammation [48, 49]. In other words, dysbiosis increases gut permeability to bacterial products and aggravates IR, facilitating systemic bacterial translocation and hepatic inflammation. As shown above, *GP* treatment improved gut barrier integrity, reduced inflammation, and alleviated endotoxemia and IR in NALFD rats. These beneficial effects might result from specific gut microbiome changes.

High-quality experimental and some human studies have confirmed the therapeutic effects of prebiotics and probiotics on NAFLD, via modulation of the gut microbiota [50]. However, no gut microbiota modulation by *GP* has been reported to date. Previous studies have found that HFD increases the ratio of *Firmicutes* to *Bacteroidetes* in NALFD rats [51]. The present study confirmed these findings and demonstrated that *GP* might exert anti-NAFLD effects by lowering the *Firmicutes*-to-*Bacteroidetes* ratio. Besides, the abundance levels of several beneficial bacteria such as *Lactococcus spp.* were increased by *GP*; in contrast, the abundance levels of pathogenic bacteria, such as *Ruminococcus spp.*, were decreased. *Lactococcus* was found to be reduced by HFD, and is negatively correlated with NAFLD [52, 53]. *Ruminococcus* ferments complex carbohydrates such as cellulose, pectin, resistant starch etc. in some cases could be pro-inflammatory [54]. Meanwhile, this study showed that gut microbiota diversity was notably recovered, suggesting that *GP* may exert hepatoprotective effects mainly by changing the *Firmicutes*-to-*Bacteroidetes* ratio, while changing the amounts of several bacterial species. However, since the host genotype can also influence gut microbiota structure, it may not be appropriate to apply these results directly to humans.

## Conclusions

Overall, this work suggests that structural changes in the gut microbiome after treatment with the Chinese herbal Medicine *GP* contribute to NAFLD amelioration. Specifically, *GP* administration resulted in enriched beneficial bacteria and suppressed pathogenic bacteria in the gut. Although it remains uncertain whether *GP*-associated gut microbiome alterations directly contribute to improving liver structure and function in NAFLD, this study provides necessary evidence demonstrating the involvement of the gut microbiota. Further studies are needed to clarify the detailed mechanisms. One of them could be faecal transplant. The effect of transfer of gut microbiota from *GP*-administered rats to NAFLD rats without any treatments should be analyzed to prove the significant association between the suppressive effect of *GP* on NAFLD and the changes in the composition of gut microbiota. Collectively, our results show *GP* as a potential microecological modulator for treating NAFLD.

## Supplementary information

**Additional file 1: Table S1.** *GP* prevents HFD-induced Non-alcoholic fatty liver disease (NAFLD) in rats **Table S2.** *GP* prevents HFD-induced inflammation in rats. **Table S3.** *GP* prevents HFD-induced inflammation in rats. **Table S4.** *GP* prevents HFD-induced insulin resistance in rats. **Table S5.** Primer sequences for DNA sequencing.

## Data Availability

The datasets used and/or analysed during the current study are available from the corresponding author on reasonable request.

## Abbreviations

ALT: Glutamic-pyruvic transaminase
AST: Glutamic oxalacetic transaminase
CVD: Cardiovascular disease
DLPC: Dilinoleoyl phosphatidylcholine
FBG: Fasting blood glucose
FINS: Fasting serum insulin
*GP*: *Gynostemma pentaphyllum*
HDL-C: High density lipoprotein cholesterol
HOMA-IR: Homeostasis model assessment of insulin resistance
IL-1β: Interleukin-1-beta
IL-6: Interleukin-6
IR: Insulin resistance
LDL-C: Low density lipoprotein cholesterol
MetS: Metabolic syndrome
NAFLD: Nonalcoholic fatty liver disease
NASH: Non-alcoholic steatohepatitis
T2D: Type 2 diabetes
TC: Total cholesterol
TG: Triglycerides
TLR-4: Toll-like receptor 4
TNF-α: Tumor necrosis factor-alpha
WB: Western blotting
