# Supplementary material for: Structural modulation of gut microbiota during alleviation of non-alcoholic fatty liver disease with Gynostemma pentaphyllum in rats
Source: BMC Complement Med Ther. 2020 Feb 5;20:34. doi: 10.1186/s12906-020-2835-7 (PMC7076883; doi:10.1186/s12906-020-2835-7)
Supplement: Supplementary file 1 — Additional file 1: Table S1. GP prevents HFD-induced Non-alcoholic fatty liver disease (NAFLD) in rats Table S2. GP prevents HFD-induced inflammation in rats. Table S3. GP prevents HFD-induced inflammation in rats. Table S4. GP prevents HFD-induced insulin resistance in rats. Table S5. Primer sequences for DNA sequencing. [file 12906_2020_2835_MOESM1_ESM.doc]

**Supplementary materials**

**Sup. Table 1. *GP* prevents HFD-induced Non-alcoholic fatty liver disease (NAFLD) in rats**

|  | ALT | AST |
| --- | --- | --- |
| NAFLD | 121.22±15.99 | 111.3±12.47 |
| Control | 38.32±6.97** | 60.30±8.64** |
| DLPC | 97.47±19.75* | 100.36±18.03 |
| GPL | 77.78±13.93** | 75.21±11.95** |
| GPM | 75.09±19.58** | 73.19±15.60** |
| GPH | 56.78±8.70** | 66.99±14.22** |

Comparison of serum concentrations of ALT and AST in each group with NAFLD group. Data are presented as mean ± standard deviation. * and ** represents statistically significant results (*P*<0.05, *P*<0.01 respectively) based on Newman–Keuls post hoc one-way ANOVA analysis.

**Sup. Table2. *GP* prevents HFD-induced inflammation in rats.**

|  | TNF-α (pg/ml) | IL-6 (pg/ml) | IL-1β (pg/ml) |
| --- | --- | --- | --- |
| NAFLD | 74.37±9.47 | 39.36±2.56 | 616.35±70.76 |
| Control | 46.38±2.04** | 22.10±4.23** | 294.96±35.80** |
| DLPC | 66.11±8.73 | 35.64±4.44 | 616.86±103.20 |
| GPL | 57.80±6.53** | 35.83±3.46 | 500.01±86.74* |
| GPM | 56.08±6.41** | 27.31±2.57** | 494.32±57.18** |
| GPH | 55.50±4.93** | 30.06±3.70** | 414.59±52.82** |

Comparison of serum concentrations of TNF-α, IL-1β and IL-6 in each group with NAFLD group. Data are shown as mean ± standard deviation. * and ** represents statistically significant results (*P*<0.05, *P*<0.01, respectively) based on Newman–Keuls post hoc one-way ANOVA analysis.

**Sup. Table 3. *GP* prevents HFD-induced inflammation in rats.**

|  | TNF-α | IL-6 | IL-1β |
| --- | --- | --- | --- |
| NAFLD | 43.59±9.39 | 14.51±2.80 | 17.12±2.26 |
| Control | 1.27±0.33** | 1.23±0.46** | 1.85±0.51** |
| DLPC | 38.42±5.94 | 13.25±3.07 | 16.05±3.67 |
| GPL | 29.63±3.67* | 7.94±0.83** | 9.95±1.71* |
| GPM | 25.45±3.97* | 7.08±0.93** | 7.70±1.11** |
| GPH | 8.54±3.12** | 4.16±0.45** | 5.94±2.02** |

The relative expression of TNF-α, IL-1β and IL-6 in each group in hepatic tissues was assessed using RT-qPCR and compared with NAFLD group. Data are shown as mean ± standard deviation. * and ** represents statistically significant results (*P*<0.05, *P*<0.01, respectively) based on Newman–Keuls post hoc one-way ANOVA analysis.

**Sup. Table 4. *GP* prevents HFD-induced insulin resistance in rats.**

|  | INS (mU/L) | GLU(mmol/L) | HOMA-IR |
| --- | --- | --- | --- |
| NAFLD | 115.39±17.70 | 9.94±1.27 | 51.11±10.48 |
| Control | 68.52±10.93** | 8.14±0.37** | 24.78±4.16** |
| DLPC | 103.05±15.86 | 9.35±2.13 | 43.51±14.39 |
| GPL | 91.88±10.70* | 8.67±1.59 | 35.42±7.67* |
| GPM | 60.16±13.49** | 7.13±1.01** | 19.16±5.79** |
| GPH | 53.39±14.26** | 7.17±0.92** | 17.33±6.13** |

Serum concentration of INS, GLU and HOMA-IR in each group was compared with NAFLD group. Data are shown as mean ± standard deviation. * and ** represents statistically significant results (*P*<0.05, *P*<0.01, respectively) based on Newman–Keuls post hoc one-way ANOVA analysis.

**Sup. Table 5. Primer sequences for DNA sequencing.**

| Primer Name | Direction | Sequence (5'to3') |
| --- | --- | --- |
| TLR4 | F | CCGCTCTGGCATCATCTTCAT |
| TLR4 | R | AAGGCTTTTCCATCCAACAGG |
| TNF-α | F | GGGCAGGTCTACTTTGGAGTCATTG |
| TNF-α | R | GGGCTCTGAGGAGTAGACGATAAAG |
| IL-1β | F | CCCAACTGGTACATCAGCACCTCTC |
| IL-1β | R | CTATGTCCCGACCATTGCTG |
| IL-6 | F | GATTGTATGAACAGCGATGATGC |
| IL-6 | R | AGAAACGGAACTCCAGAAGACC |
| GAPDH | F | TGGTGAAGGTCGGTGTGAAC |
| GAPDH | R | GCTCCTGGAAGATGGTGATGG |
